# Brain-Based Binary Communication Using Spatiotemporal Features of fNIRS Responses

**DOI:** 10.3389/fnhum.2020.00113

**Published:** 2020-04-15

**Authors:** Laurien Nagels-Coune, Amaia Benitez-Andonegui, Niels Reuter, Michael Lührs, Rainer Goebel, Peter De Weerd, Lars Riecke, Bettina Sorger

**Affiliations:** ^1^Department of Cognitive Neuroscience, Maastricht University, Maastricht, Netherlands; ^2^Maastricht Brain Imaging Center, Maastricht, Netherlands; ^3^University Psychiatric Centre Sint-Kamillus, Bierbeek, Belgium; ^4^Institute of Systems Neuroscience, Heinrich-Heine University, Düsseldorf, Germany; ^5^Institute of Neuroscience and Medicine (INM-7), Research Centre Jülich, Jülich, Germany; ^6^Brain Innovation B.V., Maastricht, Netherlands; ^7^Maastricht Centre for Systems Biology (MaCSBio), Maastricht University, Maastricht, Netherlands

**Keywords:** functional near infrared spectroscopy (fNIRS), brain computer interface, mental imagery, mental drawing, motor imagery, spatial navigation, binary communication, yes/no decoding

## Abstract

“Locked-in” patients lose their ability to communicate naturally due to motor system dysfunction. Brain-computer interfacing offers a solution for their inability to communicate by enabling motor-independent communication. Straightforward and convenient in-session communication is essential in clinical environments. The present study introduces a functional near-infrared spectroscopy (fNIRS)-based binary communication paradigm that requires limited preparation time and merely nine optodes. Eighteen healthy participants performed two mental imagery tasks, mental drawing and spatial navigation, to answer yes/no questions during one of two auditorily cued time windows. Each of the six questions was answered five times, resulting in five trials per answer. This communication paradigm thus combines both spatial (two different mental imagery tasks, here mental drawing for “yes” and spatial navigation for “no”) and temporal (distinct time windows for encoding a “yes” and “no” answer) fNIRS signal features for information encoding. Participants’ answers were decoded in simulated real-time using general linear model analysis. Joint analysis of all five encoding trials resulted in an average accuracy of 66.67 and 58.33% using the oxygenated (HbO) and deoxygenated (HbR) hemoglobin signal respectively. For half of the participants, an accuracy of 83.33% or higher was reached using either the HbO signal or the HbR signal. For four participants, effective communication with 100% accuracy was achieved using either the HbO or HbR signal. An explorative analysis investigated the differentiability of the two mental tasks based solely on spatial fNIRS signal features. Using multivariate pattern analysis (MVPA) group single-trial accuracies of 58.33% (using 20 training trials per task) and 60.56% (using 40 training trials per task) could be obtained. Combining the five trials per run using a majority voting approach heightened these MVPA accuracies to 62.04 and 75%. Additionally, an fNIRS suitability questionnaire capturing participants’ physical features was administered to explore its predictive value for evaluating general data quality. Obtained questionnaire scores correlated significantly (*r* = -0.499) with the signal-to-noise of the raw light intensities. While more work is needed to further increase decoding accuracy, this study shows the potential of answer encoding using spatiotemporal fNIRS signal features or spatial fNIRS signal features only.

## Introduction

Active human communication depends fully on the functional integrity of the motor system. When the motor system ceases to function, e.g., due to neuromuscular impairments, consequences can be detrimental for communication. Severe motor paralysis most often occurs through infarction of the pons (Patterson and Grabois, 1986) or in late stages of diseases such as amyotrophic lateral sclerosis (ALS) and multiple sclerosis. In some cases this leads to a state of being fully awake and aware (Laureys, 2005; Monti et al., 2009) but without any ability to communicate in a natural way, commonly referred to as the “locked-in” syndrome (LIS; see Plum and Posner, 1982; Laureys, 2005; Monti et al., 2009). In “classical” LIS, vertical eye movements and eye blinking are spared and can thus be used for basic communication. Nevertheless, in progressive motor-neuron disorders such as ALS, control of the eye muscles is lost in late stages of the disease, resulting in a “complete” or “total” LIS (Bauer et al., 1979).

In LIS patients, voluntarily evoked brain signals can be exploited to restore basic communication independent of motor function. This can be achieved through a brain-computer interface (BCI), which relies on intentionally generated brain signals that are measured with a functional neuroimaging method, e.g., electroencephalography (EEG; Farwell and Donchin, 1988; Leuthardt et al., 2004), magnetoencephalography (MEG; Mellinger et al., 2007; Reichert et al., 2017) or functional magnetic resonance imaging (fMRI; Sorger et al., 2009, 2012; Monti et al., 2010; Bardin et al., 2011; Naci and Owen, 2013). A BCI then processes these inputs such that they can be used for motor control, communication, neurofeedback, *etc*.

EEG is the most widely used neuroimaging method for BCI purposes. Encouraging communication results have been reported using EEG-based BCIs (Farwell and Donchin, 1988; Birbaumer et al., 1999; Leuthardt et al., 2004; Nijboer et al., 2008). Recent binary communication paradigms established accuracies consistently above 70% (Halder et al., 2010; Käthner et al., 2015), even reaching an accuracy of 87.5% in one patient (Han et al., 2019). EEG-based BCIs have been mainly tested with visual paradigms using event-related potentials that, at least partly, dependent on patients ability to fixate (Brunner et al., 2010; Treder and Blankertz, 2010). However, the population of LIS patients is heterogeneous, with varying degrees of visual impairment/oculomotor control (Riccio et al., 2012), cognitive impairment (Schnakers et al., 2008; Wilson et al., 2011) and brain areas affected. Given this patient heterogeneity, a wide range of neuroimaging methods should be explored as each has its limitations. In a recent hybrid EEG-functional near-infrared spectroscopy (fNIRS) study (Rezazadeh Sereshkeh et al., 2019) it was found that the EEG signal was detrimental in most healthy participants. Nevertheless, certain participants truly benefited from use of the fNIRS signal. The high spatial resolution of hemodynamic neuroimaging, such as fMRI and fNIRS, combined with the – typically used – auditorily guided imagery paradigms might be beneficial for certain BCI users.

A seminal fMRI paradigm (Monti et al., 2010) enabled binary communication in disorders of consciousness patients through the use of tennis imagery for encoding a “yes” response and spatial navigation imagery for a “no” response. In the 16 healthy control subjects, a decoding accuracy of 100% was obtained. Work from our lab has extended this type of paradigm to a four-choice BCI (Sorger et al., 2009), with an average accuracy of 94.9% (theoretical chance level being 25%), and a free-letter spelling BCI (Sorger et al., 2012), with an average accuracy of 82% (theoretical chance level: ca. 3.7%), successfully tested in healthy participants. Given the immobility of fMRI hardware, the current challenge is to transfer these fMRI-based paradigms to a mobile setup employing fNIRS, thereby enabling convenient BCI-based communication of patients in daily-life settings, e.g., in their home environments.

The use of fNIRS as a method to measure brain signals is gaining popularity, with the number of publications increasing rapidly (Boas et al., 2014) since its first use in 1993 (Chance et al., 1993; Hoshi and Tamura, 1993; Kato et al., 1993; Villringer et al., 1993). The mobility of fNIRS hardware makes it highly suited for bedside testing (Cutini et al., 2012; León-Carrión and León-Domínguez, 2012), hence its growing use in brain-computer interfacing (Zephaniah and Kim, 2014). However note that its mobility comes at the cost of a generally lower accuracy compared to fMRI-based paradigms. The reason for these relatively lower classification accuracies in fNIRS-based paradigms is threefold: (1) fNIRS possesses an inherently lower spatial resolution than fMRI (2) fNIRS has a limited spatial coverage and is thus only able to sample superficial regions of the cortex (3) fMRI has a higher signal-to-noise ratio (SNR) than fNIRS (Cui et al., 2011), as fNIRS suffers from global/physiological noise from extracranial tissue (Zhang et al., 2016).

In the context of communication, most hemodynamic BCI systems rely on mental imagery for intentional generation of brain signals. Commonly used mental imagery tasks (see Naseer and Hong, 2015, for a more extensive review) include mental speech (Sorger et al., 2012; Rezazadeh Sereshkeh et al., 2018), mental calculation/counting (Naito et al., 2007; Power et al., 2012; Sorger et al., 2009, 2012) and motor imagery (Coyle et al., 2007; Sorger et al., 2009, 2012; Monti et al., 2010; Abdalmalak et al., 2017a). Most fNIRS based-BCI communication studies focus on binary communication, as multi-class fNIRS-based BCIs are not yet enabling effective BCI control (Power et al., 2012; Weyand and Chau, 2015).

In most binary fNIRS-communication paradigms, a “yes” answer is encoded through mental imagery, whereas a “no” answer is encoded by rest (Abdalmalak et al., 2017b; Nagels-Coune et al., 2017; Naito et al., 2007; Naseer et al., 2014). In healthy subjects, group average accuracies range between 62 and 82% (Naseer et al., 2014; Nagels-Coune et al., 2017). In a subset of 23 out of 40 patients, an average accuracy above 75% was found using tasks activating prefrontal cortex such as mental calculation or mental singing (Naito et al., 2007). Recently Abdalmalak et al. (2017b) asked a LIS patient to imagine playing tennis for encoding a “yes”, while resting to encode a “no”. An accuracy of 100% was reached over five repetitions of three questions. The drawback from previously mentioned studies is that one cannot distinguish a real “no” answer from possible disengagement from the task. This problem can be circumvented through the use of a different, active mental task for each answer option. The evoked spatially different brain-activation patterns can then be exploited for encoding two answer alternatives. Several studies have demonstrated the potential of spatial discernibility of mental tasks using fNIRS. For example, Sitaram et al. (2007) were able to distinguish left- from right- hand motor imagery with an accuracy of 73% using a support vector machine (SVM) classification. Furthermore, Hong et al. (2015) could distinguish mental calculation, right- and left-hand imagery with an accuracy of 75.6% using 3-class linear discriminant analysis (LDA). However, to our knowledge, no study has tested the use of two mental tasks directly in a communication experiment. In a recent study, participants imagined different mental speech content for answering yes/no questions intuitively, i.e., imagining saying “yes” or “no” repeatedly (Rezazadeh Sereshkeh et al., 2018). An average accuracy of 64.1% was attained over two experimental sessions. Note, however, that only a 3-class (“yes”, “no” and “rest”) accuracy was reported, thus the 2-class accuracy (“yes” vs. “no”) cannot be inferred from the report.

The current study aimed to increase the feasibility and success of an fNIRS-BCI in healthy participants, thereby potentially increasing the applicability in LIS patients. We used an approach that combines temporal encoding (distinct time windows for encoding “yes” and “no”) with spatial encoding (two channels, each coding for a distinct mental imagery task, here motor imagery for “yes” and spatial navigation for “no”), as has been done in Sorger et al. (2009, 2012) in fMRI-based communication BCIs. In mental drawing trials, participants were asked to imagine drawing small geometric shapes with their right hand. In spatial navigation trials, participants imagined walking through their home and visualized the visual scene in different rooms. Similar tasks were previously used in the seminal fMRI work of Monti et al. (2010) and have been suggested to be explored in the context of fNIRS-BCI (Abdalmalak et al., 2017a). We expected motor cortex activation during motor imagery (Sitaram et al., 2007), and parietal activation during spatial navigation imagery (Cabrera and Dremstrup, 2008; McKendrick et al., 2016; Abdalmalak et al., 2017a). To increase general fNIRS-BCI feasibility by decreasing setup time, we opted for a sparse fNIRS optode setup with nine optodes covering large parts of left-hemispheric fronto-parietal cortex.

The current study included 18 healthy participants who were briefly trained prior to undergoing the fNIRS recording session. Participants were asked six binary questions (e.g., “Do you have a driver’s license?”) which they answered by performing one of the two tasks in auditorily cued time windows. Conventional univariate analyses were employed in simulated real-time to decode the participants’ answers from the recorded fNIRS data. Additionally, a multivariate approach was applied to explore the discernibility of the two tasks based on their spatial brain activation patterns only. Comfort ratings were obtained throughout the experiment as other studies have reported that participants may withdraw from fNIRS recordings due to headset discomfort (Suzuki et al., 2010; Cui et al., 2011; Rezazadeh Sereshkeh et al., 2018). In addition, we evaluated whether the presence of specific physical features of participants (e.g., hair thickness, root density, or color) affected fNIRS-signal quality and subsequent decoding results (Koizumi et al., 1999; Coyle et al., 2005; Cui et al., 2011; Khan et al., 2012; Fang et al., 2018). To this end, an in-house questionnaire was administered. Moreover, participants’ experience with the mental tasks in terms of ease and pleasantness were assessed, since they are known to positively correlate with decoding accuracy (Weyand and Chau, 2015).

In summary, due to the unique combination of temporal and spatial encoding of mental tasks, and the use of an active mental task for each answer option, we expected that our paradigm would outperform the standard paradigms reviewed here.

## Materials and Methods

### Participants

The current dataset was collected in the same session as the data published in a previous study by Nagels-Coune et al. (2017). Eighteen of the twenty participants performed the present paradigm in addition to the previously reported paradigm. The localizer runs (see sections Localizer Block 1 and Localizer Block 2) have already been used in the context of the earlier study (Nagels-Coune et al., 2017). All eighteen healthy participants (eight females, age = 26.00 ± 8.19 years [mean ± SD]) reported normal hearing. The participants’ characteristics of relevance to the fNIRS measurements are shown in Table 1. Written informed consent was acquired from each participant before the experiment. The experimental procedure conformed to the Declaration of Helsinki and was approved by the local ethics committee. All participants were compensated with a gift voucher for their participation.

**TABLE 1:** Participant characteristics: Handedness, capsize (in cm) and fNIRS suitability score (max score = 21).

**Participant**	**Handedness**	**Cap size**	**fNIRS suitability score**	**Mental drawing COI**		**Spatial navigation COI**	
				**HbO**	**HbR**	**HbO**	**HbR**
				
01	Right	56	12	CP3-CP5	CP3-CP5	FC3-FC1	CP3-CP5
02	Right	56	14	FC3-FC1	C3-C1	C3-FC5	FC3-FC5
03	Right	56	13	FC3-FC5	CP3-CP5	CP3-CP5	FC3-C5
04	Right	56	10	C3-FC5	FC3-C5	FC3-C5	FC3-C5
05	Left	56	10	C3-FC5	FC3-FC1	CP3-CP5	CP3-CP5
06	Right	56	16	CP3-C5	C3-C5	CP3-C5	CP3-C5
07	Right	56	14	FC3-FC5	FC3-FC5	FC3-FC5	FC3-FC5
08	Right	56	1	C3-FC5	C3-FC5	FC3-C1	C3-FC1
09	Right	56	13	C3-CP5	CP3-CP1	C3-FC5	CP3-C1
10	Right	56	14	C3-C5	C3-CP5	FC3-FC1	FC3-FC1
11	Right	56	10	FC3-C5	CP3-CP1	CP3-CP5	C3-C1
12	Right	58	17	FC3-FC5	FC3-FC5	FC3-FC5	FC3-FC1
13	Right	56	13	C3-C1	C3-C1	FC3-FC5	FC3-FC5
14	Right	58	14	CP3-CP1	C3-C5	C3-C5	C3-C5
15	Right	60	17	C3-C5	C3-C5	FC3-FC5	C3-C1
16	Right	56	10	CP3-CP1	C3-C5	FC3-FC5	C3-C1
17	Left	56	13	FC3-FC5	CP3-CP5	FC3-FC1	FC3-FC1
18	Left	56	13	C3-CP1	C3-C1	FC3-C5	FC3-C5

*The last four columns show the channels-of-interest (COIs), selected on the basis of the data of localizer runs in block 1. Abbreviations: HbO, oxygenated hemoglobin; HbR, deoxygenated hemoglobin.*

### Participant Preparation

#### Introducing the Two Mental Tasks

Following the informed consent procedure, participants were introduced to two mental imagery tasks. For the mental drawing (MD) task, participants were instructed to imagine drawing simple geometric shapes with their right hand. The three left-handed participants were thus requested to imagine drawing with their non-dominant hand. For the spatial navigation (SN) task, participants imagined walking through their house while vividly visualizing the visual scene of each room (see Supplementary Material for the standardized mental task instructions). Participants chose objects they would like to imagine drawing and a familiar environment they would like to imagine navigating through.

#### Selection of Binary Questions

Prior to the experiment, participants answered 45 unobtrusive binary questions (see Supplementary Material), e.g., “Do you have a driver’s license?” in a questionnaire. Six questions, three answered with “yes” and three answered with “no”, were selected for the main fNIRS experiment to ensure an equal distribution of both answers.

#### fNIRS Suitability Questionnaire

Due to fNIRS being an optical neuroimaging method, participants’ physical features may alter the penetration/absorption of light and consequently signal strength (Coyle et al., 2005). To evaluate whether this influenced our results, we created an in-house questionnaire that quantifies participants’ suitability for fNIRS measurements. The questionnaire (see Supplementary Material) captured the following physical features that are thought to influence fNIRS signal strength via distortion of optical contact between the skin and optodes (distortion skin-optode contact) or via light absorption: hair length (distortion skin-optode contact), hair color (light absorption; Koizumi et al., 1999; Coyle et al., 2005; Lloyd-Fox et al., 2010; Khan et al., 2012), hair thickness (light absorption affected by hair follicle density; Coyle et al., 2005; Fang et al., 2018), hair density (distortion skin-optode contact; Lloyd-Fox et al., 2010; Orihuela-Espina et al., 2010), hair structure (distortion skin-optode contact; Lloyd-Fox et al., 2010); skin color (light absorption by melanin concentration; Orihuela-Espina et al., 2010), and head size (light absorption affected by altered inter-optode distance). Each feature was rated on a scale ranging from 0 (desirable feature) to a maximum of 4 (undesirable feature). Scores were summed with a maximum score of 21. The higher the suitability score, the less suitable for fNIRS measurement the participant was deemed.

#### Cap Placement and Mental Task Training

Participants’ head circumference was measured and an appropriately sized cap was selected. Cap sizes used in this experiment ranged from 54 to 60 cm (see Table 1). Prior to placing the cap, participants were asked to moisten the left side of the head to aid the placement of the optodes. Similar to EEG cap placement, nasion-inion distance was measured to ensure proper cap positioning. Participants were then seated in a sound-attenuating cabin, which was kept entirely dark during the fNIRS measurement as ambient light can influence near-infrared spectroscopy measurements (Kovalenko et al., 2014; Pinti et al., 2018). While the optodes were placed in optode holders, participants were given the opportunity to practice the two mental tasks. This procedure took on average 17 min (standard deviation: ± 8 min).

### fNIRS-Based Communication Paradigm

We employed an auditorily cued encoding paradigm in which fNIRS signals were evoked through differently timed (temporal encoding) mental imagery tasks (spatial encoding). The auditory cues, i.e., concise spoken commands, guided participants’ mental imagery by indicating the start and end of each encoding window. The cues and their accompanying time point triggers were presented using an in-house software (StimulGL; Gijsen, 2015). Our design encompassed two localizer runs (block 1), six encoding runs, and finally another two localizer runs (block 2).

#### Localizer Block 1

In the two localizer runs, participants performed the two tasks in a fixed order, with the MD run preceding the SN run. These localizer runs were conducted to gauge participant’s hemodynamic responses to the mental tasks and select task-sensitive channels for answer decoding. In the first localizer run, participants performed 20 MD trials with a duration of 10 s each, interleaved with 20 s rest periods. The localizer started with an initial rest period of 20 s, after which the participants heard the auditory cue “start”. This cue marked the start of the MD task, by which participants were instructed to perform the mental imagery task until they heard the cue “rest”. They then halted the mental imagery and remained at rest for 20 s, until the next “start” cue urged them to commence the mental imagery again. This procedure was repeated 20 times, resulting in 20 MD trials. The second localizer followed the same protocol.

#### Six Answer Encoding Runs

**FIGURE 1:**
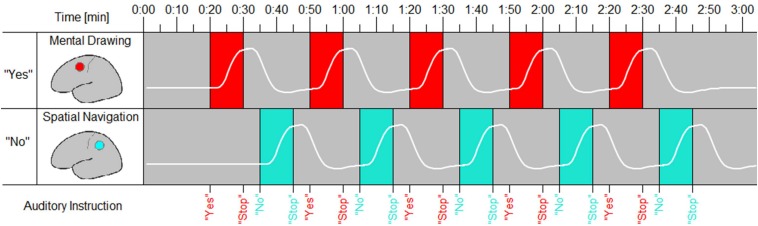
Encoding scheme for answering a binary question. The red periods require mental drawing (MD) imagery, whereas the green periods required spatial navigation (SN) imagery. If participants chose to answer “yes”, they started performing the MD task when they heard a “yes”, halted their imagery when they heard the cue “stop”, and ignored the auditory cues related to the “no” response. The hypothesized HbO response for a “yes” answer is shown by the upper white waveform. If participants chose to answer “no”, they started performing the SN task when they heard a “no”, halted their imagery when they heard the cue “stop”, and ignored the auditory cues related to the “yes” response. The hypothesized HbO response for a “no” answer is shown by the lower white waveform.

In this stage of the experiment, participants were asked to answer binary questions by performing one of two mental tasks in a particular time window. Participants were informed that to encode a “yes” answer, they had to perform MD imagery when they heard “yes”. In the “yes” encoding runs, participants were instructed to ignore the “no” cues and to not perform SN (or any other task). Conversely, to encode a “no” answer, they had to perform SN imagery when they heard “no”. In this case, the “yes” cues were ignored (see Figure 1).

Six questions were asked, each at the start of a separate answer encoding run. The question was read aloud through a microphone by the experimentor. The fNIRS recording started when the participant reported having his/her answer, i.e., “yes” or “no”, and the corresponding task, i.e., MD or SN, clearly in mind. The time intervals within which a “yes” or “no” answer could be given followed 20 s after termination of the question. The “yes” interval was initiated by the auditory “yes” cue and terminated 10 s later by an auditory “stop” cue. The “yes” interval always proceeded the “no” interval, which was marked by the auditory cue “no” and “stop” 10 s later. Hence, if participants had chosen to answer “yes”, they started performing the MD. If participants had chosen to answer “no”, they ignored the “yes” cue. The cue, “stop”, indicated participants to stop the mental imagery. Again, if participants had chosen to answer “no”, they also ignored this “stop” cue and remained at rest until they heard the cue “no”. At this point they started performing the SN task until the “stop” cue was heard. This procedure was repeated five times per encoding run, resulting in five “yes” and five “no” trials.

Summarized, participants thus answered questions by performing the MD mental task within a first time interval marked by “yes” and “stop” cues, or the SN mental task within a second time interval marked by “no” and “stop” cues.

#### Localizer Block 2

The procedure in localizer block 1 was repeated to increase the amount of available data for classifier training. This repetition was warranted, as we were unsure with respect to the minimum amount of data necessary for effectively training the classifier in the multivariate approach.

### Comfortability Ratings

In between the ten fNIRS runs, participants were allowed to take a short break to slightly adjust their body posture, or drink some water. After each run, participants were asked to give a comfortability rating between 0 and 10, with 0 meaning “very uncomfortable” and 10 being “very comfortable”.

### Ease and Pleasantness of the Mental Tasks

After the ten fNIRS runs, the cap was removed and participants were asked to rate the ease and pleasantness of the MD and SN tasks with a score from 0 to 10. An easiness rating of 0 indicated great difficulty of mental task execution, whereas a rating of 10 indicated extreme ease of task execution. A pleasantness rating of 0 indicated an extremely unpleasant experience when performing the mental task, whereas a score of 10 indicated an extremely pleasant experience.

### fNIRS Data Acquisition

**FIGURE 2:**
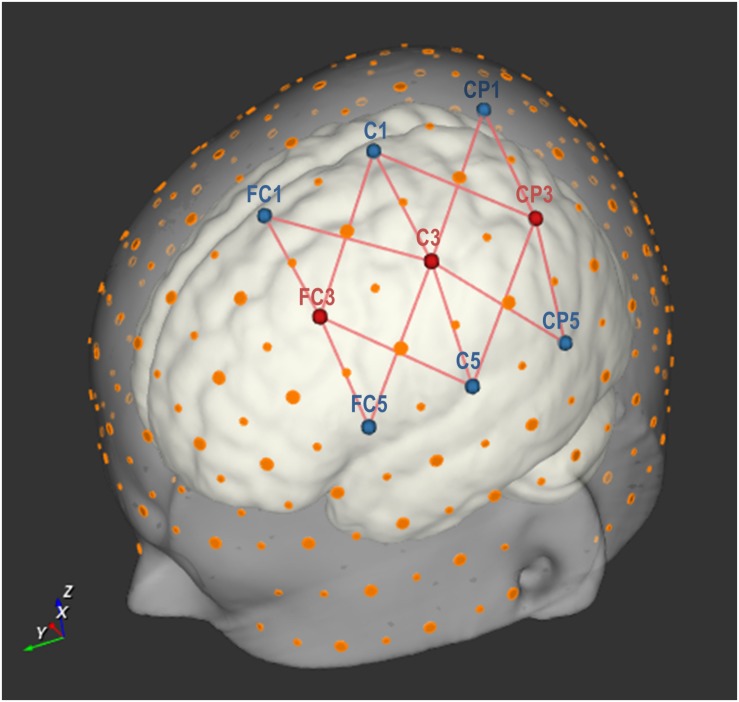
Optode setup with three source and six detector optodes, placed on nine points according to the international 10-20 EEG system. Large orange dots represent reference points of the 10-20 system, whereas small orange dots represent reference points of the extended 10-10 EEG system (Oostenveld and Praamstra, 2001). The red lines represent 14 source-detector pairs (each forming an fNIRS channel). Image created using NIRSite (v.1) software (NIRx Medizintechnik GmbH, Berlin, Germany; RRID: SCR_002491).

Hemodynamic signals were obtained using a continuous-wave fNIRS system (NIRScout-816 system, NIRx Medizintechnik GmbH, Berlin, Germany; RRID: SCR_002491) and NIRStar (v. 12.0) software (NIRx Medizintechnik GmbH, Berlin, Germany; RRID: SCR_014540). Three source optodes, LEDs emitting light with wavelengths of 760 and 850 nm, were used in combination with six detector optodes. These nine optodes were placed in optode holders on the cap according to the international 10-20 EEG system. The three sources optodes were positioned on FC3, C3, and CP3, whereas the six detector optodes were positioned on FC5, C5, CP5, FC1, C1, and CP1 (see Figure 2). Defining a channel as a unique source and detector optode pair, this setup resulted in 18 channels. Channels FC3-CP1, FC3-CP5, CP3-FC1, and CP3-FC5 were excluded from all analyses, as the spatial separation between the sources and detectors exceeded 60 mm in the largest cap size (60 cm) used in this experiment. An optode separation of that size was considered undesirable since it largely exceeds the recommended inter-optode distance of 30 mm and gives rise to noisy and unstable signals (Gratton et al., 2006). The remaining 14 channels analyzed in this experiment are depicted by the red connecting lines in Figure 2. This optode montage covered a confined area above the left-hemispheric fronto-parietal (sensorimotor) cortex. The frontal optodes covered brain areas commonly associated with motor imagery, such as premotor cortex and possibly parts of the supplementary motor areas in certain head sizes (Sitaram et al., 2007; Koessler et al., 2009; Abdalmalak et al., 2016). The posterior optodes captured part of the parietal cortex, expected to be associated with SN imagery (Cabrera and Dremstrup, 2008; McKendrick et al., 2016; Abdalmalak et al., 2017a). Optical signals were recorded with a sampling rate of 12.5 Hz.

### Data Analysis

#### Analyses of the fNIRS Signal

**FIGURE 3:**
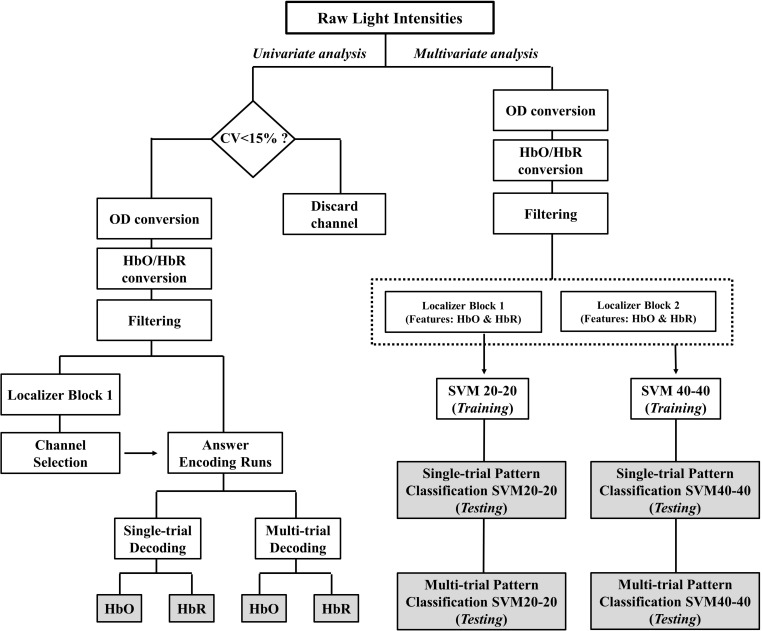
Schematic depiction of the fNIRS signal analyses. The two main pipelines were univariate analysis and multivariate analysis. Each pipeline resulted in four accuracy outcomes. These outcome variables are represented in gray colored boxes. CV, coefficient of variance; OD, optical density; HbO, oxygenated hemoglobin; HbR, deoxygenated hemoglobin; SVM20-20, support vector machine with 20 training trials of each task; SVM40-40, support vector machine with 40 training trials of each task.

The main outcome of the spatiotemporal encoding paradigm, i.e., communication accuracy, was obtained with a General Linear Model (GLM) approach (univariate analysis). In addition, spatial discernibility of the two mental tasks was investigated using a SVM (multivariate analysis). See Figure 3 for an illustration of the analyses workflow.

##### General data (pre)-processing

FNIRS time series were analyzed in simulated real-time using Turbo-Satori software (v1.2.8, Brain Innovation B.V., Maastricht, Netherlands). In the first pre-processing step, raw wavelengths were converted to optical densities. The optical density data were then converted to oxygenated hemoglobin (HbO) and deoxygenated hemoglobin (HbR) values using the modified Beer-Lambert law. Linear trend removal and moving average filtering (low-pass cut-off frequency: 0.3 Hz, filter order: 2; high-pass cut-off frequency: 0.01 Hz, filter order: 1) were applied. The low-pass filter aimed to remove high-frequency artifacts induced by heartbeat and breathing, whereas the high-pass filter served to remove low-frequency drifts.

##### Communication accuracy (univariate analysis)

###### Channel exclusion

To ensure proper signal quality, we excluded channels showing a signal-to-noise ratio below a given criterion value. To that aim, the channel-wise coefficient of variance percentage (CV%) was calculated on the unfiltered raw wavelength data by dividing the temporal standard deviation by the mean value (see Piper et al., 2014, for a detailed description). A CV% higher than 15 indicates insufficient signal-to-noise ratio (Schmitz et al., 2005; Schneider et al., 2011; Piper et al., 2014; Pfeifer et al., 2018). Consequently, all channels with a CV% higher than 15 for either one or both wavelengths in the first two localizers (block 1) were excluded from channel-of-interest selection. This channel-wise exclusion was also performed on the last set of localizers (block 2) to gauge the intra-individual variability in channel exclusion across all localizer runs.

Given the limited number of 14 channels in the current experiment, one runs the risk of excluding a potentially informative channel due to its high CV%. Therefore, all univariate analyses were repeated omitting the CV% criterion, thus allowing different channels to be selected for subsequent analyses (see Supplementary Material). Only when the overall accuracy differed significantly between both approaches, the accuracies of the analyses without the CV% criterion are also reported in the results section.

###### Channel selection

From the channels that were not excluded in the previous step, a single channel was chosen for each mental imagery task (MD and SN) based on the data of localizer block 1. HbO and HbR signals were analyzed separately. Four GLM analyses (HbO/HbR × MD/SN) were conducted with a predictor for the mental imagery trials and applying the contrast “MD/SN vs. rest”. The four channels with the highest *t*-value in each of the four GLM analyses were coined “channels of interest” (COIs) and were considered for the following single- and multi-trial analyses of the answer encoding data.

###### Answer decoding

Participant’s answers were decoded through comparison of the five individual trial pairs (single-trial approach) or through comparison of the integrated five trials per answer option (multi-trial approach).

In the single-trial approach a GLM analysis was run with the statistical contrast “yes” vs. “no” for each yes/no trial pair (5 trial pairs per encoding run). This resulted in four *t*-values per trial pair, one for each COI (HbO/HbR × SN/MD), based on mental task predictors that encompassed two individual trials. These *t*-values were used to decode the participants’ answer as follows: When the *t*-value of the MD COI was larger than the *t*-value of the SN COI, a “yes” answer was decoded. Whereas when the *t*-value of the MD COI was smaller than the *t*-value of the SN COI, a “no” answer was decoded. The single-trial approach resulted in 30 decoded answers (6 runs × 5 trial pairs) per participant. The decoded answers were compared to the originally encoded answers and each individual participant’s accuracy was calculated.

In the multi-trial approach, a GLM analysis was run with the statistical contrast “yes” vs. “no”. The five trials per answer option were used to infer a *t*-value for each COI (HbO/HbR × SN/MD). Decoding followed the same rationale of *t*-value comparison as in the single-trial approach. This procedure was repeated for all six answer-encoding runs for both the HbO and HbR signal separately. The decoded answers were compared to the originally encoded answers and each individual participant’s accuracy was calculated. The multi-trial approach resulted in 6 decoded answers (6 runs) per participant. The decoded answers were compared to the originally encoded answers and each individual participant’s accuracy was calculated.

To assess the significance of the participants’ decoding accuracies in the univariate analyses, we determined the empirical chance level based on binomial distributions (Noirhomme et al., 2014). The following settings were determined: α = 0.05, number of independent outcomes *k* = 2 and number of independent trials *n* = 30 or *n* = 6 for single- and multi-trial accuracies respectively. The resulting upper-bound empirical chance levels for evaluating single- and multi-trial accuracies were therefore 19 trials (63.33%) and 5 trials (83.33%) respectively. If 19 or more trials were decoded correctly in the single-trial approach, this was considered a significant result. If 5 or more trials were decoded correctly in the multi-trial approach, this was considered a significant result.

The rate of correct detection of “yes”/“no” answers was calculated by dividing the amount of correctly detected “yes”/“no” answers by the total amount of encoded “yes”/“no” answers per participant, i.e., 15 for the single-trial and 3 for the multi-trial analysis.

##### Multivariate analyses

###### Single-trial results

Two classifiers were trained to discriminate the spatial activation patterns in all 14 channels induced by the two different mental tasks. This was done using either 20 or 40 trials of each mental task. One classifier was trained on two runs: one for MD (MD1) and one for SN (SN1), with each 20 trials (SVM20-20). The other classifier was trained on four runs: two for MD (MD1 and MD2) and two for SN (SN1 and SN2), resulting in 40 trials for each task (SVM40-40). We considered a temporal window spanning -2 s to 20 s (where 0–10 s corresponds to the trial interval) and linearly fitted each HbO/HbR concentration channel time course separately with a design matrix consisting of a double-gamma hemodynamic response function per trial and an additional linear confound predictor. Resulting estimates were *t*-values which were stored in volume map files for each time course. These files were used as input for the classifier testing on an independent dataset, i.e., the six answer-encoding runs’ data. Per answer-encoding run, the five “active” trials, i.e., trials in which we knew the participant was performing a task, were tested. The five “inactive” trials in which participants rested were not analyzed. This resulted into a total of thirty testing trials (6 runs × 5 “active” trials) per participant. The proportion of testing trials for which the decoded answer matched the true answer was subsequently calculated. Lastly, to determine the empirical chance level for each individual participant, permutation testing was performed with an in-house MATLAB script (ver. R2015a). To this end, task labels were randomly reassigned to each trial in the training dataset, on which the classifier was subsequently trained. Testing was then done on an independent, non-permuted testing dataset. This procedure was repeated 2000 times. Chance level was calculated as the proportion of permutations revealing accuracies lower or equal to the accuracy obtained using the real (non-permuted) dataset.

The rate of correct detection of MD/SN was calculated by dividing the amount of correctly detected MD/SN patterns by the total amount of encoded MD/SN trials, i.e., 15 per task.

###### Multi-trial results

Multi-trial accuracies were derived from the single-trial multivariate results reported above. The five yes/no decisions per run were integrated using majority voting (e.g., three answers encoded as “yes” and two answers encoded as “no” were considered as a “yes” answer, and vice versa). The proportion of decoded answers matching with the true answer was calculated for each participant. The upper-bound empirical chance level for each individual participant was 83.33%, based on binomial distributions.

The rate of correct detection of MD/SN was calculated by dividing the amount of correctly detected MD/SN patterns by the total amount of encoded MD/SN runs, i.e., 3 per task.

#### fNIRS Suitability Questionnaire and Signal Quality

The total fNIRS suitability score was obtained by summing all features, with a maximum score of 21 (see Table 1 for suitability score per participant). This score was correlated with the number of channels with a CV under 15%, which is a metric for fNIRS signal quality (Balardin et al., 2017), through calculation of a one-tailed Pearson’s *r* in SPPS (ver. 22). Furthermore linear regression analyses were performed using SPPS (ver. 22). FNIRS signal quality (i.e., SNR) was treated as predictor variable and the eight decoding accuracies obtained from the univariate analyses (single-/multi-trial × HbO/HbR) and multivariate analyses (single-/multi-trial × SVM20-20/SVM40-40) were treated as criterion variables.

#### Comfortability, Ease and Pleasantness Ratings

Mean and standard deviation are reported for comfortability, ease and pleasantness ratings. Pearson’s *r* was calculated between ease and pleasantness and accuracy outcomes of all univariate (single-/multi-trial × HbO/HbR) and multivariate (single-/multi-trial × SVM20-20/SVM40-40) analyses. Statistical significance was evaluated using a criterion of α = 0.05.

## Results

### Communication Accuracy (Univariate Analysis)

#### Channel Exclusion

On average 37% of channels were excluded due to their low SNR in localizer block 1. Descriptively, the channels with a relatively longer source-detector distance, e.g., diagonal channels such as FC3-C1, as compared to the shorter optode distances, e.g., straight channels such as FC3-FC1, were excluded more often. Large variation was observed between individual participants, ranging from 0 to 13 excluded channels. In contrast, the SNR measure was highly consistent across the four localizer runs (block 1 and block 2) within individual participants (see Supplementary Figure S1).

#### Channel Selection

**FIGURE 4:**
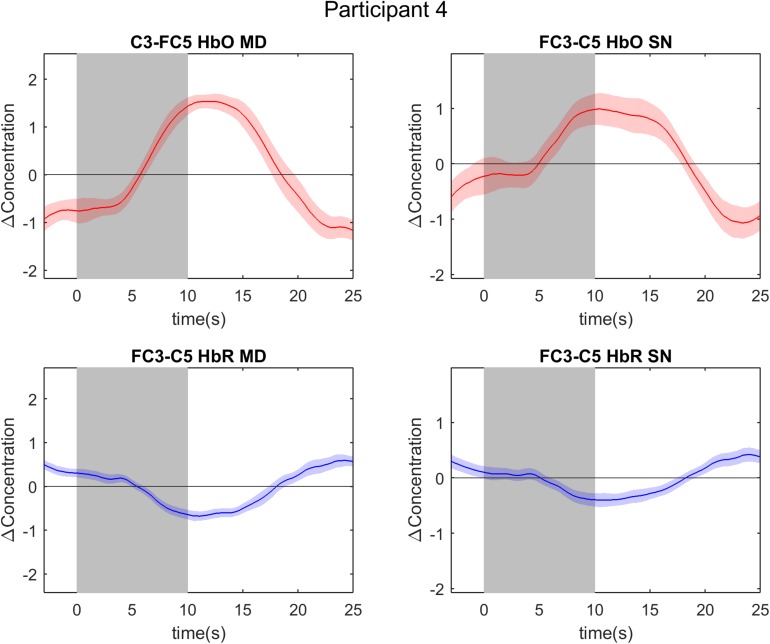
Event-related averages of channels of interest in participant 4. The two graphs on the left are event-related averages from the first localizer run (mental drawing; MD). The two graphs on the right are event-related averages from the second localizer run (spatial navigation; SN). The top two graphs depict the oxygenated hemoglobin (HbO) response, whereas the bottom two graphs depict the deoxygenated hemoglobin (HbR) response. Each graph is the event-related average of 20 individual trials, with the darker average signal line and its standard deviation (lighter colored band surrounding the average signal line). Notice the clear and typical hemodynamic response during both tasks: a positive deflection in HbO and a negative deflection in HbR. The gray band from 0 to 10 s signifies the mental imagery time interval.

**FIGURE 5:**
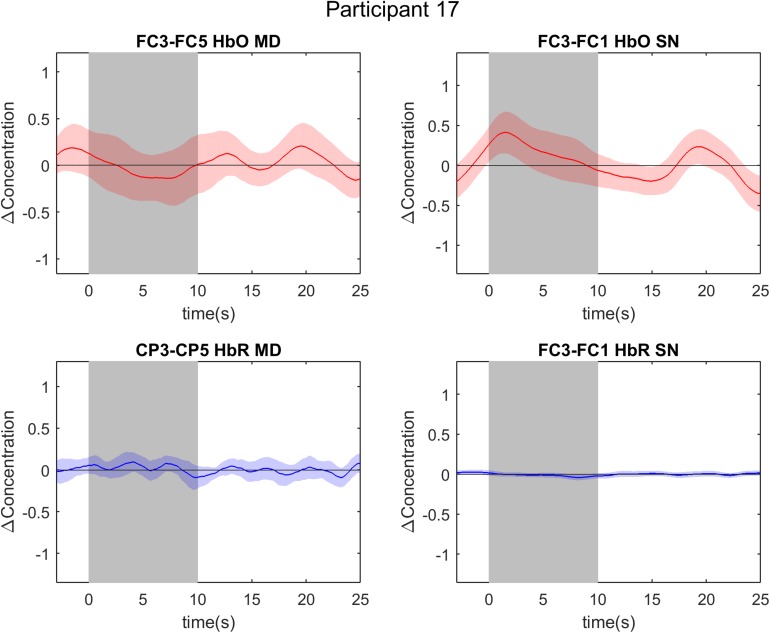
Event-related averages of channels of interest in participant 17. The two graphs on the left are event-related averages from the first localizer run (mental drawing; MD). The two graphs on the right are event-related averages from the second localizer run (spatial navigation; SN). The top two graphs depict the oxygenated hemoglobin (HbO) response, whereas the bottom two graphs depict the deoxygenated hemoglobin (HbR) response. Each graph is the event-related average of 20 individual trials, with the darker average signal line and its standard deviation (lighter colored band surrounding the average signal line). Notice the absence of a typical hemodynamic response during both tasks: there is no clear positive deflection in HbO, nor a negative deflection in HbR. The gray band from 0 to 10 s signifies the mental imagery time interval.

The four COIs selected for further data-analysis steps are reported per participant in Table 1. In the HbO selection, the same channel was selected for mental drawing and spatial navigation imagery in three participants, i.e., participant 6, 7, and 12. In the HbR selection the same channel was selected for both tasks in four participants, i.e., participant 1, 4, 7, and 14. Overall the channel selection was quite variable across participants (see Table 2). For the MD task, channels FC3-FC5 (HbO) and C3-C5 (HbR) were chosen most frequently. For the SN task, channels FC3-FC5 (HbO) and FC3-FC5, FC3-C5, FC3-CP1, and C3-CP3 (HbO) were chosen most frequently. The event-related averages of the four channels-of-interest are depicted for two exemplary participants, participant 4 and 17 (Figures 4, 5).

**TABLE 2:** Frequency table of channel-of-interest selection in 18 participants.

**Channel (source-detector) l**	**Absolute frequency**			
	**Mental drawing**		**Spatial navigation**	
	**HbO**	**HbR**	**HbO**	**HbR**
FC3-FC5	4	2	5	3
FC3-C5	1	1	2	3
FC3-FC1	1	1	3	3
FC3-C1	0	0	1	0
C3-FC5	3	1	2	0
C3-C5	2	4	1	1
C3-CP5	1	1	0	0
C3-FC1	0	0	0	1
C3-C1	1	3	0	3
C3-CP1	1	0	0	0
CP3-C5	1	0	1	1
CP3-CP5	1	3	3	2
CP3-C1	0	0	0	1
CP3-CP1	2	2	0	0
Total	18	18	18	18

*A channel is formed by the combination of two optodes (source-detector). Abbreviations: HbO, oxygenated hemoglobin; HbR, deoxygenated hemoglobin.*

#### Single-Trial Results

**FIGURE 6:**
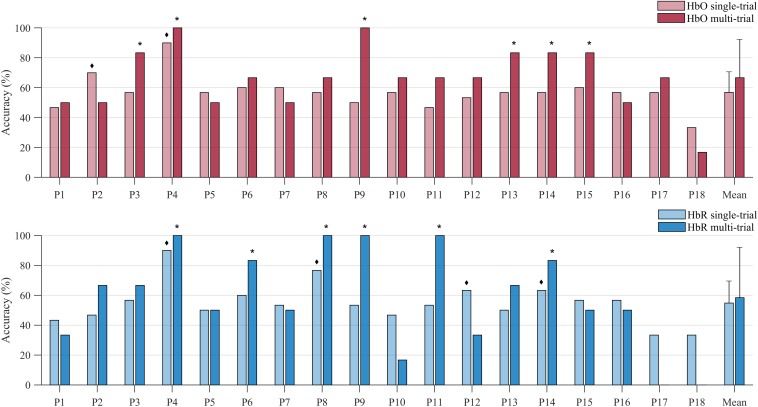
Decoding accuracies of individual participants and the sample mean obtained with the single-trial (light-colored bars) and the multi-trial (dark-colored bars) univariate approach. Decoding accuracies were attained through channels-of-interest, preceded by a channel exclusion step. The upper plot show results based on analysis of HbO data (red bars), the lower plot is based on HbR data (blue bars). The ◆ symbol indicates participants whose single-trial accuracy was significant, whereas the * symbol indicates those participants whose multi-trial accuracy was significant.

Univariate analysis of single-trial data resulted in an average decoding accuracy of 56.85% (*SD* = 11.17%) and 54.81% (*SD* = 13.58%) for HbO and HbR respectively (see Figure 6). Individual accuracies ranged from 33.33 to 90%. Two participants’ HbO data decoding accuracy was significant (indicated with a ◆ symbol in Figure 6). The average rate of correct detection of “yes” answers in the HbO signal was 60.00%, whereas “no” answers were correctly detected 53.70% of the time. The HbR decoding accuracy was significant in four participants (indicated with a ◆ symbol in Figure 6). Participant 4 was the sole significant participant in both HbO and HbR accuracies. The average rate of correct detection of “yes” answers in the HbR signal was 62.22%, whereas “no” answers were correctly detected 47.41% of the time.

#### Multi-Trial Results

**FIGURE 7:**
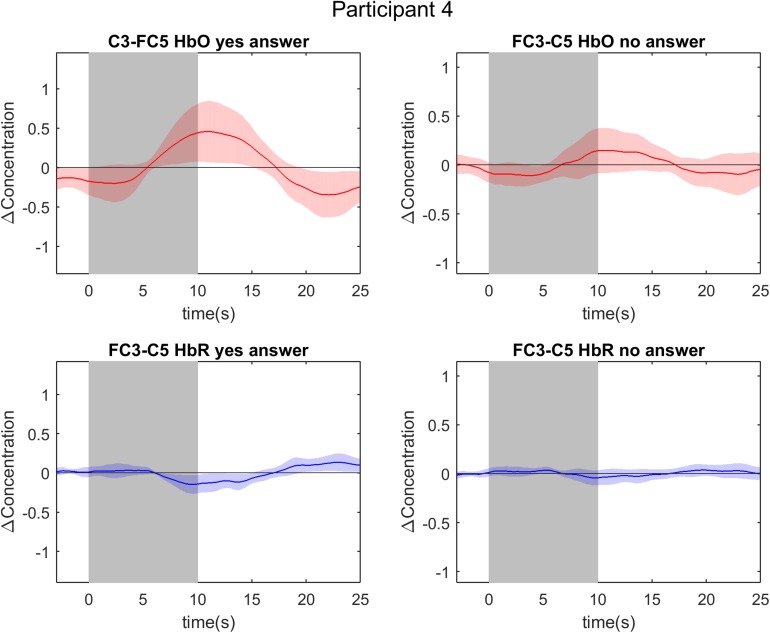
Event-related averages of channels-of-interest in participant 4. The two graphs on the left are event-related averages from the first answer decoding run, in which the participant encoded a “yes” answer. The two graphs on the right are event-related averages from the sixth answer decoding run, in which the participant encoded a “no” answer. The top two graphs depict the oxygenated hemoglobin (HbO) response, whereas the bottom two graphs depict the deoxygenated hemoglobin (HbR) response. Each graph is the event-related average of five individual trials, with the darker average signal line and its standard deviation (lighter colored band surrounding the average signal line). Notice the clear and typical hemodynamic response function during both tasks: a positive deflection in HbO and a negative deflection in HbR. The gray band from 0 to 10 s signifies the mental imagery time interval.

**FIGURE 8:**
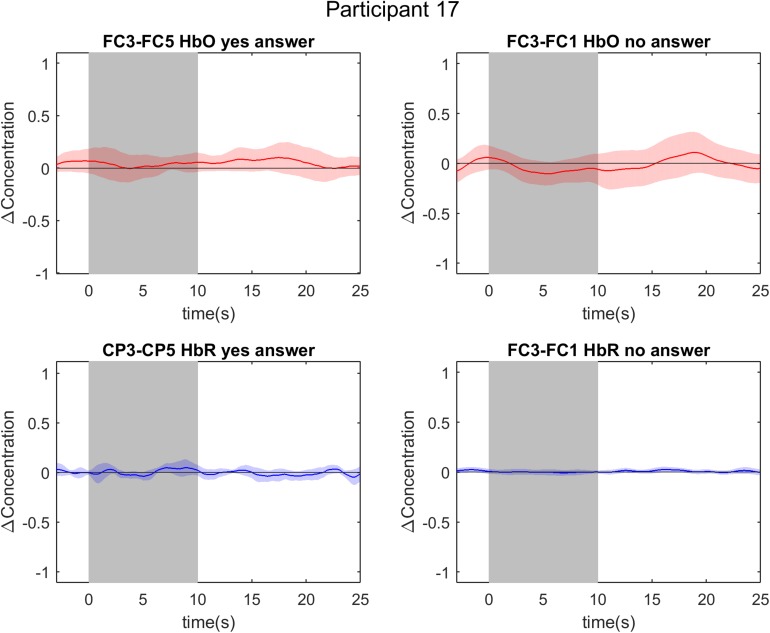
Event-related averages of channels-of-interest in participant 17. The two graphs on the left are event-related averages from the fifth answer decoding run, in which the participant encoded a “yes” answer. The two graphs on the right are event-related averages from the sixth answer decoding run, in which the participant encoded a “no” answer. The top two graphs depict the oxygenated hemoglobin (HbO) response, whereas the bottom two graphs depict the deoxygenated hemoglobin (HbR) response. Each graph is the event-related average of five individual trials, with the darker average signal line and its standard deviation (lighter colored band surrounding the average signal line). Notice the absence of a typical hemodynamic response function during both tasks: there is no clear positive deflection in HbO, nor a negative deflection in HbR. The gray band from 0 to 10 s signifies the mental imagery time interval.

Univariate analysis of multi-trial decoding resulted in an average accuracy of 66.67% (*SD* = 20.6%) and 58.33% (*SD* = 32.96%) for HbO and HbR respectively (see Figure 6). The control analysis without channel exclusion yielded a significantly lower group average of 58.33% (*SD* = 25.73%) for the HbO data (paired samples *t*-test; *t* = 2.70; *p* = 0.015 (see Supplementary Material and Supplementary Figure S2). In the main analysis, i.e., with channel exclusion, individual accuracies ranged from 0 to 100%. Six participants’ HbO data decoding accuracy was found to be significant (indicated with a ^∗^ symbol in Figure 6). The answers by participants 4 and 9 were decoded with 100% accuracy. The average rate of correct detection of “yes” answers in the HbO signal was 83.33%, whereas “no” answers were correctly detected in 50.00% of the cases. The HbR decoding accuracy was significant in six participants (indicated with a ^∗^ symbol in Figure 6), with 100% accuracy in participants 4, 8, 9, and 11. The answers of participants 4, 9, and 14 were significantly decoded in both HbO and HbR signal. For illustrative purposes the event-related averages of a “yes” and a “no” answer are depicted for participant 4 and 17 in Figures 7, 8. The average rate of correct detection of “yes” answers in the HbR signal was 72.22%, whereas “no” answers were correctly detected 44.44% of the time.

### Multivariate Analyses

#### Single-Trial Results

**FIGURE 9:**
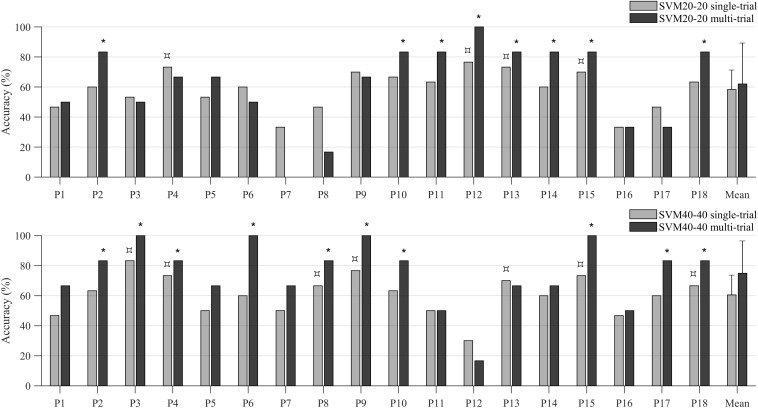
Decoding accuracies of individual participants and the sample mean obtained with the single-trial (light-colored bars) and the multi-trial (dark-colored bars) multivariate approach. The upper plot shows decoding accuracies of the SVM20-20 classifier, the lower plot shows decoding accuracies of the SVM40-40 classifier. The 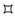 symbol indicates participants whose accuracy reached significance, as tested with permutation testing (for evaluating single-trial accuracies), whereas the * symbol indicates those participants whose multi-trial accuracy was significant. Abbreviations: SVM20-20 = support vector machine with 20 training trials of each task; SVM40-40 = support vector machine with 40 training trials of each task.

The SVM20-20 classifier achieved an accuracy of 58.33% (*SD* = 13.05%). Individual accuracies ranged from 33.33 to 76.67%. Spatial activation patterns could be distinguished significantly above chance level, assessed by permutation testing, in four out of 18 participants (indicated with a 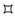 symbol in the top plot in Figure 9). The average rate of correct detection of MD was 52.59%, whereas SN was correctly detected 64.07% of the time.

The SVM40-40 classif ier achieved an accuracy of 60.56% (*SD* = 13.15). Individual accuracies ranged from 30.00 to 83.33%. Spatial activation patterns could be distinguished significantly above chance level, assessed by permutation testing, in seven out of 18 participants (indicated with a 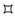 symbol in the bottom plot in Figure 9). The average rate of correct detection of MD was 59.26%, whereas SN was correctly detected 62.59% of the time.

#### Multi-Trial Results

The SVM20-20 classifier achieved an accuracy of 62.04% (*SD* = 27.30%). Individual accuracies ranged from 0 to 100%. Spatial activation patterns discernibility was significant in eight out of 18 participants (indicated with a ^∗^ symbol in the top plot in Figure 9). The average decoding accuracy of these eight participants was 85.42% (*SD* = 5.89%). The average rate of correct detection of MD was 59.26%, whereas SN was correctly detected 64.81% of the time.

The SVM40-40 classifier achieved an accuracy of 75.00% (*SD* = 21.58%). Individual accuracies ranged from 16.67 to 100%. Spatial activation patterns discernibility was significant in ten out of 18 participants (indicated with a ^∗^ symbol in the bottom plot in Figure 9). The average rate of correct detection of MD was 72.22%, whereas SN was correctly detected 77.78% of the time.

### fNIRS Suitability Questionnaire and Signal Quality

We found that SNR, as measured by the number of channels passing the CV% criterion, was significantly correlated with fNIRS suitability scores (*r* = -0.499, *n* = 18, *p* = 0.018; see Supplementary Figure S3). Participants with low fNIRS suitability scores (indicating highly suitable participants) thus typically had good fNIRS signal SNR.

Regression analyses with accuracy as the criterion variable and SNR as the predictor variable revealed the following results (see Supplementary Figure S4). Approximately 31% of the variation in HbR multi-trial accuracy could be attributed to the variation in SNR (*R*^2^ = 0.309 with *F*_17_ = 7.165, *p* = 0.017). In contrast, SNR was no significant predictor for any other criterion variable: HbR single-trial accuracy (*R*^2^ = 0.083 with *F*_17_ = 1.442, *p* = 0.247), HbO single-trial accuracy (*R*^2^ = 0.045 with *F*_17_ = 0.755, *p* = 0.398), HbO multi-trial accuracy (*R*^2^ = 0.029 with *F*_17_ = 0.480, *p* = 0.499), single-trial SVM20-20 (*R*^2^ = 0.135 with *F*_17_ = 2.494, *p* = 0.134), multi-trial SVM20-20 (*R*^2^ = 0.041 with *F*_17_ = 0.676, *p* = 0.423), single-trial SVM40-40 (*R*^2^ = 0.009 with *F*_17_ = 0.144, *p* = 0.709) and multi-trial SVM40-40 (*R*^2^ = 0.147 with *F*_17_ = 2.767, *p* = 0.116).

### Comfortability, Easiness and Pleasantness

**FIGURE 10:**
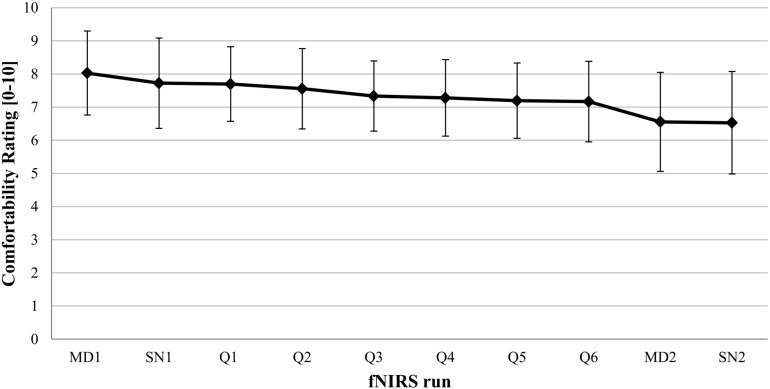
Mean comfortability rating over time (fNIRS runs). A comfortability rating of 0 corresponds to “very uncomfortable” and 10 to “very comfortable”. The ten fNIRS runs are depicted in the order they were conducted in the experiment. The first two runs, MD1 and SN2, were localizer runs (block 1) for mental drawing (MD) and spatial navigation (SN). The following six runs, Q1, Q2, Q3, Q4, Q5, Q6, represent the answer decoding runs, with a Q as an abbreviation for “question run”. The last two runs, MD2 and SN2, were localizer runs (block 2). Error bars reflect standard deviations.

Participant’s comfortability rating started out fairly high (8.03 ± 1.27) and then decreased over the remaining fNIRS runs (see Figure 10). The last run shows lowered although still acceptable comfort scores (6.53 ± 1.55). Not a single participant indicated a comfortability score lower than 5 during the experiment.

Overall both tasks were deemed easy and pleasant. On average the SN task was considered more difficult to perform (6.28 ± 1.32) and less pleasant (6.61 ± 1.45) than the MD task (7.94 ± 1.48; 7.28 ± 1.49). The difference between the two tasks in terms of ease was statistically significant (*t* = 4.70, *p* < 0.001). The difference in pleasantness showed a similar trend, yet it was not statistically significant (*t* = 1.86, *p* = 0.081). Ease and pleasantness ratings correlated significantly with the accuracy of the SVM40-40 analysis, whereas all other correlations were not significant (see Table 3 for all correlations).

**TABLE 3:** Correlation table of ease and pleasantness ratings with the eight accuracy outcomes variables.

**Pearson’s p *p*-value**	**Multi-trial HbO**	**Multi-trial HbR**	**Single-trial HbO**	**Single-trial HbR**	**Single-trial SVM 20-20**	**Multi-trial SVM 20-20**	**Single-trial SVM 40-40**	**Multi-trial SVM40-40**
Ease MD	0.288, 0.123	0.220, 0.190	0.101, 0.345	0.034, 0.447	0.010, 0.484	0.017, 0.945	0.508, 0.016*	0.383, 0.117
Ease SN	0.360, 0.071	0.147, 0.281	0.156, 0.268	0.041, 0.435	0.325, 0.094	0.364, 0.137	0.609, 0.004*	0.499, 0.035*
Pleasantness MD	0.320, 0.098	0.210, 0.202	0.127, 0.308	−0.051, 0.421	−0.116, 0.323	−0.208, 0.408	0.736, 0.000*	0.748, 0.000*
Pleasantness SN	0.295, 0.117	0.023, 0.464	0.023, 0.464	−0.163, 0.259	0.145, 0.283	0.076, 0.765	0.637, 0.002*	0.423, 0.080

Abbreviations: MD, mental drawing; SN, spatial navigation; HbO, oxygenated hemoglobin; HbR, deoxygenated hemoglobin; SVM, support vector machine; * = correlation is significant at 0.05 level (1-tailed).

## Discussion

We presented a novel binary communication paradigm that aimed to exploit spatiotemporal characteristics of fNIRS signals evoked by differently timed mental imagery tasks. The paradigm involved minimal training and a sparse optode setup of only nine optodes (three sources, six detectors). Participants were asked to perform mental drawing (MD) for encoding a “yes” answer and spatial navigation (SN) for encoding a “no” answer in different auditorily cued time windows. The applied goal was to test decoding success and feasibility of the current paradigm compared to previous paradigms. Answers were decoded in simulated real-time using a set of predefined fNIRS channels and a univariate analysis approach. We also performed an explorative multivariate analysis on the data from all channels to investigate the differentiability of the two mental tasks based solely on spatial fNIRS signal features. In addition, the link between participants’ physical characteristics and the fNIRS signal was explored with a novel fNIRS suitability questionnaire.

### Univariate Analysis

#### Channel Selection

We hypothesized that relatively frontal optodes covered brain regions commonly associated with motor imagery, whereas posterior optodes covered brain areas associated with SN imagery (see section fNIRS Data Acquisition). On a group level, we found that frontal optodes were selected most often, irrespective of the type of task. However, note that a channel exclusion step was performed before the channel selection step, thus one should interpret these findings with caution. On an individual level, spatially different channels were selected as COI for each task in most participants. The absence of a spatial encoding aspect (i.e., selecting the same COI for both tasks) in a few participants (three in HbO and four in HbR; see Table 1) posed no decoding problem. Our paradigm aimed at exploiting spatial as well as temporal characteristics of fNIRS signals. Hence, in those few participants we solely relied on the temporal aspect, the fNIRS signal evoked by differently timed mental imagery tasks, to decode participant’s answers. For example, participant 4 had the same COI for both tasks in the HbR signal but had a decoding accuracy clearly above chance level, with a single-trial accuracy of 90% and a multi-trial accuracy of 100%. The incorporation of both spatial and temporal features is an experimental safeguard in the presented fNIRS paradigm.

#### Communication Accuracy

The single-trial GLM approach, with average decoding accuracies of 56.85% (HbO) and 54.81% (HbR), enabled effective communication in a limited subset of participants. In the fNIRS literature, no univariate single-trial accuracies have been previously reported. Multiple trials seem to be necessary at the current time, unfortunately at the cost of a lower information transfer rate. The multi-trial GLM approach resulted in higher group decoding accuracies in comparison to the single-trial approach. In four participants a 100% decoding accuracy was reached in the multi-trial approach, which was not attained in any participant using a single-trial approach. Average multi-trial decoding accuracy was higher in HbO (66.67%) than in HbR (58.33%), but on an individual level the same number of participants (six) reached significance. The similar individual decoding results across HbO and HbR were an unexpected finding. Generally, the lower amplitude and SNR of HbR, as compared to HbO, is thought to hinder detection of task-evoked changes (Leff et al., 2011). In line with this, it has been demonstrated that HbO signal is more robust than HbR for motor imagery specific activation (Mihara et al., 2012). Likewise, Rezazadeh Sereshkeh et al. (2018) reported that HbO signals yielded the highest accuracies in their 3-class BCI using imagined speech, and Hwang et al. (2016) reported that HbO features yield more discriminative information than HbR features in 2-class communication. Despite this previous work, here we find individual HbR multi-trial decoding accuracies that are similar to the ones seen in the HbO signal. It could be that the negative effect of the low SNR of the HbR signal is compensated by the relatively low sensitivity to physiological noise, i.e., systemic artifacts in both extra-cerebral and intra-cerebral compartments, as compared to HbO (Kirilina et al., 2012). In the current study we could not correct for physiological noise, which might have been a disadvantage for the HbO signal especially. Whether the differential sensitivity to physiological noise should influence researchers’ decision to select either HbO or HbR for BCI purposes should be investigated further. Therefore, in line with Pinti et al. (2018), we encourage future studies to report both HbO and HbR results.

As in previous fNIRS-BCI studies, only a subset of our participants reached an acceptable criterion for communication (Naito et al., 2007; Nagels-Coune et al., 2017; Rezazadeh Sereshkeh et al., 2018). The multi-trial approach enabled effective communication in six participants in the HbO signal, i.e., participants 3, 4, 9, 13, 14, 15, and six participants in the HbR signal, i.e., participants 4, 6, 8, 9, 11, and 14. When taking the HbO and HbR results together, effective communication was reached in half of our participants. Therefore, as stated above, we recommend reporting BCI success for both HbO and HbR in future studies. Note that our use of the empirical chance level as a criterion is significantly stricter than the commonly used “70%” criterion that signifies a sufficient accuracy for communication in an individual user (Kubler et al., 2006). Our paradigm thus enables effective communication, greatly exceeding the common criterion of 70% for effective communication (Kubler et al., 2006), in a subset of participants.

Our multi-trial accuracies of 66.67% (HbO) and 58.33% (HbR) are low compared to those reported in other binary communication paradigms (Naseer et al., 2014; Rezazadeh Sereshkeh et al., 2018). This could be due to our sparse approach of a single session. Other studies encompassed multiple sessions (Rezazadeh Sereshkeh et al., 2018) or separate training sessions (Naseer et al., 2014). More training of our participants and more experimental trials could have resulted in better BCI performance (Kaiser et al., 2014) but would require more time investment, which in turn might affect the clinical applicability.

Our paradigm is the first to attempt using two active mental tasks to differentiate two answer options. However the low correct detection rate of the “no” answers, ranging from 44.44 to 53.70%, implies that the motor imagery task has mainly driven our univariate results. This finding questions the effective contribution of the spatial navigation task in our univariate analyses. Efforts have been made to investigate SN in naturalistic environments (McKendrick et al., 2016) and virtual reality environments (Kober et al., 2013) using fNIRS. However, to our knowledge no previous fNIRS study has explored the fNIRS signal in response to SN imagery. This study thus constitutes the first exploration of SN imagery in fNIRS. Future studies should investigate this mental task more thoroughly using an extended optode setup, as it is possible that our optode setup was not suited for SN. Alternatively, other promising mental imagery tasks can be explored. With respect to the spatial encoding aspect of the current paradigm (two distinct mental tasks and associated channels-of-interest for encoding “yes” and “no” encoding), follow-up work is required to ensure effective and balanced contributions of both tasks.

### Multivariate Analysis

The multivariate analysis explored the possibility of distinguishing the spatial patterns induced by MD vs. SN, disregarding any temporal information. From a clinical perspective, we compared the classifier results for both a limited (localizer block 1) and a full (localizer block 1 and 2) training set. Both our single-trial decoding accuracies, 58.33% (SVM20-20) and 60.56% (SVM40-40), were rather low in comparison with previous studies. Classification results of 73% in two-class discrimination (Sitaram et al., 2007) and 64.1 to 75.6% in three-class discrimination (Hong et al., 2015; Rezazadeh Sereshkeh et al., 2018) are reported. However, the limited amount of trials in the current study should be noted, whereas other studies have trained and tested their classifiers on a significantly higher number of trials. In addition, our setup of nine optodes is quite sparse in comparison to previous work (Sitaram et al., 2007; Hong et al., 2015; Rezazadeh Sereshkeh et al., 2018). Note that the correct detection of the MD and SN tasks was more balanced, as compared to the univariate analyses. Correct detection of MD ranged from 52.59 to 72.22%, while correct detection of SN ranged from 62.59 to 77.78%. This implies effective contribution of both mental imagery tasks in our multivariate analyses.

Interestingly, in the current experiment, a simplistic majority voting approach applied on the single-trial SVM decisions, resulted in heightened accuracies of 62.04% (SVM20-20) and 75% (SVM40-40). This type of trial combination is rarely reported in BCI literature (Nagels-Coune et al., 2017), but it seems to affect the decoding accuracy in a positive manner and could potentially be useful in clinical BCI applications.

A limitation of our multivariate approach is that the two mental imagery tasks never co-occurred within localizer runs. Classifiers were thus trained on each distinctive task in one (SVM20-20) or two (SVM40-40) separate runs. In hindsight, it would have been better to perform both mental tasks within a run, as has been done by e.g., Valente et al. (2019) in an MVPA-based BCI control study using fMRI.

### Uni- vs. Multivariate Results

Comparisons between the univariate and multivariate results should be drawn with caution given the fundamentally different nature of the methods. In the univariate analyses, the data from four channels-of-interest were considered, whereas all channels were considered in the multivariate analyses. Each analysis approach has its drawbacks for future BCI use, with the SVM approach requiring more measurement points and the GLM approach being dependent on a small subset of channels. There is no clear superiority of one approach over the other and one could think of these methods as two alternatives that can be explored depending on the BCI user’s preferences and performance. Despite similar average decoding accuracies across uni- and multivariate analyses, accuracies varied largely within an individual participant. For example, the surprisingly low multi-trial decoding accuracy of 0% in HbR for participants 17 and 18 is in stark contrast with their MVPA accuracy. In Figures 5, 8, one cannot recognize the expected hemodynamic response (positive HbO deflection and negative HbR deflection) or any other response in the signal of participant 17. The 0% finding in the HbR signal for the multivariate analyses is thus probably due to noisy signal in combination with a low number of trials (6 trials), as both participants attain an accuracy of 33.33% in the single-trial analysis. In addition, suboptimal channel selection due to our sparse optode setup might have contributed to these findings. Nevertheless, when looking at the multivariate results of participant 17 and 18, we see responses above chance level. These diverging results between uni- and multivariate analyses imply that our general linear model approach, with its focus on a single channel-of-interest for each task, was not well suited to disentangle the differential spatial features of the fNIRS signal in certain participants.

### Inter-Subject Variability

The inter-subject variability in our sample was substantial, both in terms of signal quality and accuracy outcomes. The large variability between participants has been recognized in other fNIRS studies (Holper et al., 2011; Power et al., 2012; Rezazadeh Sereshkeh et al., 2018). We have explored a few subject-specific factors that potentially influence the fNIRS signal quality and accuracy, such as hair and skin features (fNIRS suitability questionnaire) and subjective ease and pleasantness ratings of the mental tasks.

#### fNIRS Suitability Questionnaire

We developed an fNIRS suitability questionnaire to explore whether physical features such as hair and skin could predict fNIRS signal quality. In the current study, we found that participants who were deemed less suitable for fNIRS (as measured by our in-house questionnaire), generally had less channels with a sufficient SNR (as operationalized by CV%). The resulting significant correlation constituted a first indicator of the questionnaire’s usefulness. Furthermore, the variation in SNR across participants could explain approximately 31% of the variance in the HbR multi-trial accuracies (*R*^2^ = 0.309 with *F*_17_ = 7.165, *p* = 0.017). Note however that the fNIRS suitability questionnaire administered in the current study is an exploratory instrument and further work is needed to establish its validity and reliability. In addition, it should be noted that we used common optode holders, as opposed to spring-loaded optode holders, in the current experiment. Common optode holders are thought to be more sensitive to signal disturbance due to hair than spring-loaded optode holders. It is thus expected that the established relationship between physical features and fNIRS signal quality will weaken in an experimental set-up with spring-loaded optode holders. However, given the participant discomfort they often cause (Lloyd-Fox et al., 2010), non-spring loaded optode holders will continue to be used in studies involving children, patients and other vulnerable populations. More extensive exploration of the effects of participants’ hair, skin and head size on signal quality is required in the future. Ideally one would determine a suitability criterion that ensures sufficient SNR and thus enables detection of intentional brain activation.

#### Comfort, Ease, and Pleasantness

Our participants generally experienced the fNIRS setup as comfortable. Despite the average decrease of comfortability across time, participants still felt comfortable in the last fNIRS runs and not a single participant indicated discomfort at any point.

Participants considered the MD significantly easier to perform than the SN. In addition the SN task was considered less pleasant than the MD task. Despite a clear trend, this difference did not reach significance. Ease and enjoyment have been shown to correlate with fNIRS decoding accuracy (Weyand and Chau, 2015). In line with these observations, ease and pleasantness correlated significantly with the SVM40-40 accuracies in the current study (see Table 3).

#### Unexplored User Characteristics

In half of our participants, the paradigm did not enable effective communication. While this may in part be due to the poor signal quality of the current data set, with on average 37% of channels rejected per participant, other studies have similarly identified subgroups of participants in which fNIRS-BCI failed to work (Naito et al., 2007; Rezazadeh Sereshkeh et al., 2018). Given the general recognition of substantial inter-subject variability, the current challenge in fNIRS-based BCI research is to investigate what enables certain participants to use the BCI successfully but also what factors are hindering BCI success in other participants. Given the known correlations between EEG-BCI success and user characteristics (Weyand and Chau, 2015), a systematic investigation of user characteristics in relation to fNIRS-BCI performance is due. Factors that are thought to influence fNIRS hemodynamic signatures are age (Zich et al., 2017), handedness (Kempny et al., 2016), user training (Kaiser et al., 2014), vividness of mental imagery (Cui et al., 2007), imagery content in combination with idiosyncratic cognitive abilities (Holper and Wolf, 2011) and mental fatigue (Sargent et al., 2018). Lastly, there is notable inter-subject variability in brain activation patterns elicited by certain mental tasks (Power et al., 2012; Weyand and Chau, 2015). Therefore, an individualized combination of two tasks may be most effective for controlling a binary BCI in individual users. A first effort to explore each participant’s best discriminating subset of mental tasks has shown encouraging results (Weyand and Chau, 2015).

### Limitations and Future Work

In the current study, three left-handed participants, i.e., participants 5, 17, and 18, were asked to perform motor imagery with their non-dominant hand. Given the established hemispheric asymmetry related to handedness (Maruff et al., 1999; Lee et al., 2019; Yokoyama et al., 2019), it is plausible that left hand imagery combined with right hemisphere fNIRS recordings would have resulted in heightened BCI decoding accuracies for these three participants. When excluding these three participants from our univariate analyses, single-trial accuracies rose to 58.44% (HbO) and 58.00% (HbR), previously 56.85 and 54.81%. Multi-trial accuracies rose to 70.83% (HbO) and 62.50% (HbR), previously 66.67 and 58.33% respectively.

The signal quality in the current data set may have been limited by our use of non-spring loaded optode holders. Recently the use of spring loaded optode holders is on the rise, as they are known to improve signal quality. Unfortunately the type of optode holders is not systematically reported in fNIRS studies, thereby limiting systematic comparison. Nevertheless, given the discomfort they often cause (Lloyd-Fox et al., 2010), non-spring loaded optode holders will continue to be used in patient studies. Therefore the current data might be representative for data we might encounter in patient population. It is known that the signal-to-noise ratio of fNIRS measurement remains a challenge in ecologically valid environments (Zephaniah and Kim, 2014; Pinti et al., 2017). Our presented fNIRS suitability questionnaire should be developed further and would ideally identify those participants with an insufficient SNR before the start of the experiment. Given this information, efforts can be made to ensure good signal quality by for example tracking the optode-to-scalp coupling in real-time (Pollonini et al., 2016).

Another drawback of the current study is the absence of additional physiological measures. Taking measures of blood pressure, respiration and heart rate (Bauernfeind et al., 2014), and regressing out these factors from our HbO and HbR signals might have improved our detection of task-specific activation. Moreover, given the absence of short-separation channels in the current study, we could not remove the influence of extra-cerebral tissue changes on the fNIRS signal (Brigadoi and Cooper, 2015). Methods such as the global component removal by Zhang et al. (2016) require optodes to cover a much larger area than the expected activated area and could thus not be applied. Mayer waves might thus have occurred in our dataset and have possibly reduced our decoding accuracies (Yucel et al., 2016). This might be especially the case for HbO as compared to HbR, given its higher sensitivity to physiological noise (Kirilina et al., 2012). Future studies should incorporate short-separation channels, as this can result in a significant improvement in both accuracy and reliability of fNIRS measurements (Brigadoi and Cooper, 2015). Such improvements are warranted for transference of fNIRS-BCI to clinical populations, as there is empirical evidence from EEG-based BCI that accuracies tend to be lower in patients as compared to healthy participants (Halder et al., 2010).

We advise future studies that employ a similar paradigm to focus on multi-trial decoding accuracies, as these proved most promising in our univariate analysis. This general linear model approach using a small set of fNIRS channels has enabled effective communication in half of our participants in either HbO or HbR signal. The good HbR decoding accuracies were an unexpected finding and we thus advise future experiments to report both HbO and HbR signal outcomes. In addition, future experiments should perform online, real-time, analysis. This would enable direct within-session feedback, which may heighten motivation in the participants and subsequently BCI performance (Kleih et al., 2010; Nijboer et al., 2010). Lastly, efforts to combine fNIRS with other modalities, such as EEG, have shown to improve classification accuracy significantly (Fazli et al., 2012; Zephaniah and Kim, 2014; Shin et al., 2018; Rezazadeh Sereshkeh et al., 2019) and are worth further investigation.

## Conclusion

The presented binary communication paradigm aimed to exploit spatiotemporal characteristics of fNIRS-signals evoked by differently timed mental imagery tasks. In various univariate analyses, the group average decoding accuracy was limited and did not exceed previously reported paradigms. The mental drawing imagery mainly drove our decoding results in the univariate analyses. Spatial navigation imagery should be explored more extensively in the context of fNIRS. Despite the rather low group average accuracies or number of participants exceeding chance level, it bears mention that those participants with a significant decoding accuracy performed excellent, with participants reaching decoding accuracies of 100%. The multivariate results showed potential spatial discernibility in a subset of participants. Integration of the single-trial multivariate outcomes using a majority voting approach resulted in encouraging decoding accuracies. The hypothesized link between participants’ physical characteristics and the fNIRS signal was confirmed with our novel fNIRS suitability questionnaire.

## Data Availability Statement

The raw data supporting the conclusions of this article will be made available by the authors, without undue reservation, to any qualified researcher.

## Ethics Statement

The study involved human participants and was reviewed and approved by the Ethische Commissie Psychologie (ECP), Faculty of Psychology and Neuroscience, Maastricht University. The participants provided their written informed consent to participate in this study.

## Author Contributions

NR and BS conceived and designed the study as well as obtained the data. LN-C performed the data analysis with the aid of AB-A, ML, and BS. BS, PD, LR, and RG oversaw the analyses. LN-C wrote the first draft of the manuscript. All authors contributed to manuscript revision, read and approved the submitted version.

## Conflict of Interest

ML and RG are employed by the research company Brain Innovation B.V., Maastricht, Netherlands.

The remaining authors declare that the research was conducted in the absence of any commercial or financial relationships that could be construed as a potential conflict of interest.

## Abbreviations

COI: channel-of-interest
MD: mental drawing
SN: spatial navigation.
