# Supplementary material for: Brain-Based Binary Communication Using Spatiotemporal Features of fNIRS Responses
Source: Front Hum Neurosci. 2020 Apr 15;14:113. doi: 10.3389/fnhum.2020.00113 (PMC7174771; doi:10.3389/fnhum.2020.00113)
Supplement: Supplementary file 1 [file Data_Sheet_1.pdf]

Supplementary Material

1 Binary Questions

Do you have a brother?

Do you own a car?

Do you sleep before 12'o clock?

Have you been beyond Europe already?

Do you like sports?

Have you ever driven a motor scooter?

Do you like listening to classical music?

Do you watch the news at 8'o clock?

Were you born in Maastricht?

Would ever like to visit the moon?

Were you born in the Netherlands?

Do you have a driver's license?

Do you eat pork?

Are you older 28 years?

Do you have any sisters?

Are you married?

Is your favorite color red?

Do you like cats?

Do you drink coffee in the morning?

Do you go on winter holidays?

Do you have children?

Do you have a smart phone?

Were you born before 1985?

Do you live in Maastricht?

Do you have a dog?

Do you live in a house?

Did you study in Germany?

Do you like spaghetti?

Do you go on summer holidays?

Do you have a laptop?

Do you miss your hometown?

Did you graduate in Cologne?

Did you visit the school in Magdeburg?

Do you want to stay forever in your current home town?

Did you have a job while going to school?

Do you like the summer season?

Did you graduate in Maastricht?

Have you ever visited USA?

Do you like the color pink?

Do you like to play football?

Did you immediately find a job after your education?

Do you like the winter season?

Do you like to play volleyball?

Do you like to swim in the sea?

Do you like your hometown?

2 Standardized Mental Task Instructions

**Mental task**	**Instruction**
Mental drawing	During 'mental drawing', please imagine drawing simple geometric figures (such as circles, triangles, cubes, *etc.*) or small contour drawings (*e.g.*, a butterfly, star, car, tree, boat, or house). Do this with your right hand and in a comfortable but consistent speed. Try to imagine using a pen. This might support your imagination. Start from the beginning if necessary.
Spatial navigation	During 'spatial navigation', you should imagine to "go" through your house/apartment and look into the different rooms for a moment (*e.g.*, 2s). Do that in a comfortable but consistent speed. Try to really imagine vividly the various three-dimensional (3D) scenes. The order of the rooms does not matter. However, try to continuously perform this mental task (thus continuously try to imagine the 3D scene of the particular room). After having looked into all rooms of your house/apartment immediately start from the beginning and perform the spatial navigation task as long as it is indicated.
Resting	During 'resting' try relax and not to do anything, especially not to perform the mental tasks implemented in this study. Try to neglect irrelevant auditory instructions (*e.g.*, when encoding the answer "No"*)*.

3 fNIRS Suitability Questionnaire

Participant ID:

Handedness: left/right

1. Hair

	Features	Values	Evaluation
Hair Length	bald (naturally)	0	
	shaved	1	
	short I (<3cm)	2	
	short II (>3cm)	3	
	long (>20cm)	4	
Hair Color	(bald)	0	
	light (blond, white)	1	
	middle (dark blond, light brown, red, grey)	2	
	dark (dark brown, black)	3	
Hair (Root) Thickness	(bald)	0	
	fine	1	
	middle	2	
	dense	3	
Hair Density	(bald)	0	
	thin	1	
	middle	2	
	thick	3	
Hair Structure	(bald)	0	
	straight	1	
	undulating	2	
	curly	3	

2. Skin

	Features	Values	Evaluation
Skin Color	light	1	
	tanned	2	
	dark	3	

3. Head

	Features	Values	Evaluation
Head Size	< 58	0	
	58/60	1	
	> 60	2	

4. Score

4 Frequency of Accepted Channels per Participant

In 11 participants the same number of channels was excluded across all four localizer runs. In the remaining 7, the maximum difference in number of excluded channels between localizer runs was one (see Figure S1).

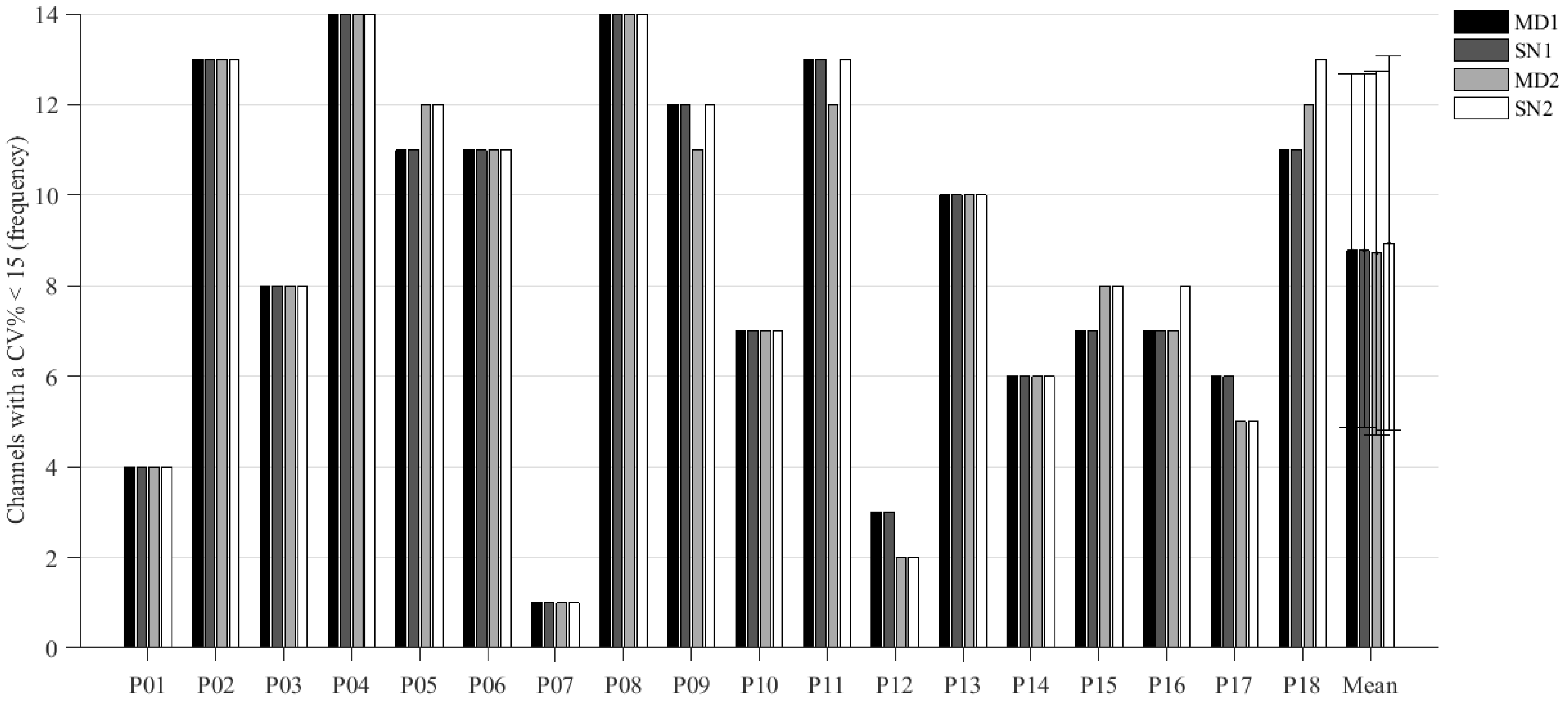

Figure S1: Number of channels with a coefficient of variance (CV%) smaller than 15, *i.e.*, channels for all participants, and the sample mean. Legend abbreviations: MD1: mental drawing localizer 1 (block1); MD2: mental drawing localizer 2 (block 2); SN1: spatial navigation localizer 1 (block 1); SN2: spatial navigation localizer 2 (block 2)**.**

5 Univariate Results without the Channel Exclusion Step

Given the limited amount of 14 channels in the current experiment, one runs the risk of excluding a potentially informative channel due to its low SNR. Therefore, all univariate analyses were repeated omitting the CV% criterion, thus allowing different channels to be selected for subsequent analyses. Only when overall accuracy differed significantly between both approaches, the accuracies of the analyses without the CV% criterion were reported in the main manuscript.

5.1 Channel Selection

In table S1 the selected channels-of-interest (COIs) can be seen per participant. When compared to table 1 (analysis with channel exclusion), there is a 58% overlap in HbO COIs and 69% in HbR COIs.

Participant	Handedness	Cap Size	fNIRS Suitability Score	Mental Drawing COIs		Spatial Navigation COIs	
				HbO	HbR	HbO	HbR
01	Right	56	12	C3-FC1	C3-FC1	C3-FC5	C3-FC5
02	Right	56	14	FC3-FC1	C3-C1	C3-FC5	FC3-FC5
03	Right	56	13	C3-CP5	CP3-CP5	CP3-CP5	FC3-C5
04	Right	56	10	C3-FC5	FC3-C5	FC3-C5	FC3-C5
05	Left	56	10	C3-FC5	FC3-FC1	CP3-CP5	C3-CP5
06	Right	56	16	CP3-C5	C3-C5	FC3-C1	CP3-C5
07	Right	56	14	CP3-CP1	CP3-CP1	C3-FC1	CP3-CP1
08	Right	56	1	C3-FC5	C3-FC5	FC3-C1	C3-FC1
09	Right	56	13	C3-CP5	CP3-CP1	C3-FC5	CP3-C1
10	Right	56	14	C3-C5	C3-CP5	FC3-FC1	C3-CP1
11	Right	56	10	FC3-C5	CP3-CP1	CP3-CP5	C3-C1
12	Right	58	17	C3-CP1	CP3-CP1	C3-CP5	C3-CP5
13	Right	56	13	CP3-C1	CP3-C1	C3-CP1	FC3-FC5
14	Right	58	14	CP3-CP1	C3-C5	C3-C5	C3-C5
15	Right	60	17	C3-C5	C3-C5	FC3-FC5	FC3-C5

16	Right	56	10	FC3-C1	FC3-C1	C3-CP5	C3-C1
17	Left	56	13	C3-CP1	C3-CP1	C3-FC1	FC3-FC1
18	Left	56	13	C3-CP1	C3-C1	FC3-C1	FC3-C5

Table S1: Participant characteristics: Handedness, capsize (in cm) and fNIRS suitability score (max score = 21). The last four columns show the channels-of-interest (COIs), selected on the basis of the data of localizer runs in block 1 without the channel exclusion step. The cells with a grey background signify the channels that overlap in analyses with and without the channel exclusion step.

5.2 Single-trial Results

Univariate analysis of single-trial data resulted in an average accuracy of 55.93 % (SD = 13.89%) and 53.33 % (SD = 14.69%) for HbO and HbR respectively. Individual accuracies ranged from 26.67% to 90%. Five participants' HbO data decoding accuracy was significant. Three participants' HbR data decoding accuracy was significant. Participant 4 was the sole participant whose answers were decoded significantly using HbO or HbR signal.

5.3 Multi-trial Results

Univariate analysis of multi-trial decoding resulted in an average accuracy of 58.33% (SD = 25.72) and 57.41% (SD = 33.93) using HbO and HbR data, respectively. Individual accuracies ranged from 0% to 100%. Four participants' HbO data decoding accuracy was significant. Seven participants' HbR data decoding accuracy were significant. The answers of participants 4, 9 and 14 were decoded significantly in both HbO and HbR signal.

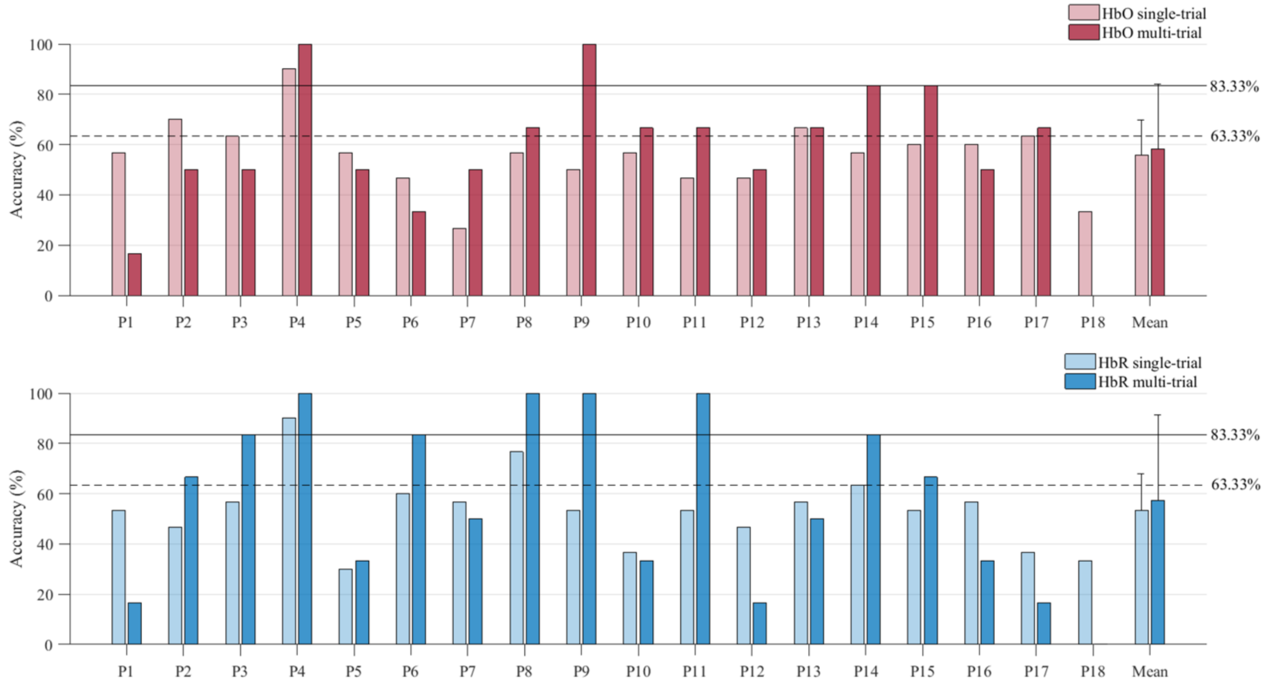

Figure S2: Decoding accuracies of individual participants and the sample mean obtained with the single-trial GLM approach (light-colored bars) and the multi-trial approach (dark-colored bars). . Decoding accuracies were attained through channels-of-interest, not preceded by a channel exclusion step. The upper plots show results based on analysis of HbO data (red bars), the lower plot is based on HbR data (blue bars). The horizontal lines represent the empirical chance levels of 63.33% (dashed line, for evaluating single-trial accuracies) and 83.33% (solid line, for evaluating multi-trial accuracies).

6 fNIRS Suitability Questionnaire and Signal Quality

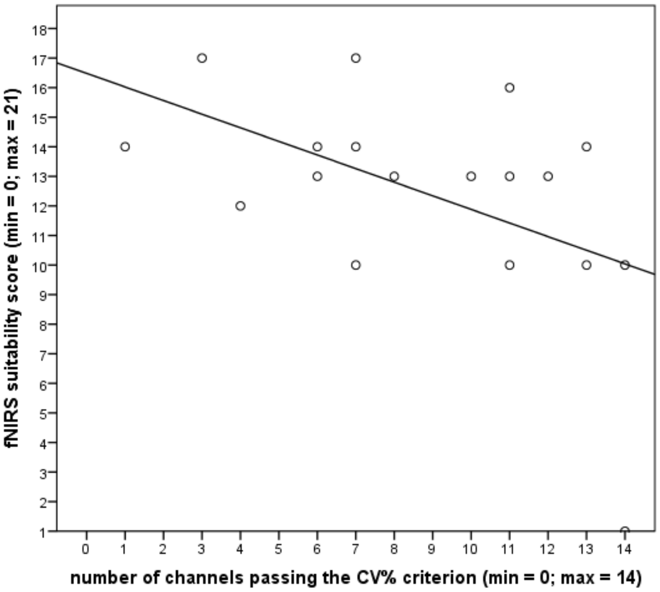

Figure S3: Scatterplot with an SNR measure on the x-axis and fNIRS suitability score on the y-axis. SNR is operationalized as the number of channels passing the CV% criterion, with a higher number indicating better SNR. Note that the higher suitability scores on the y-axis indicate possibly less suitable participants for fNIRS measurements. The solid black line is the linear regression line.

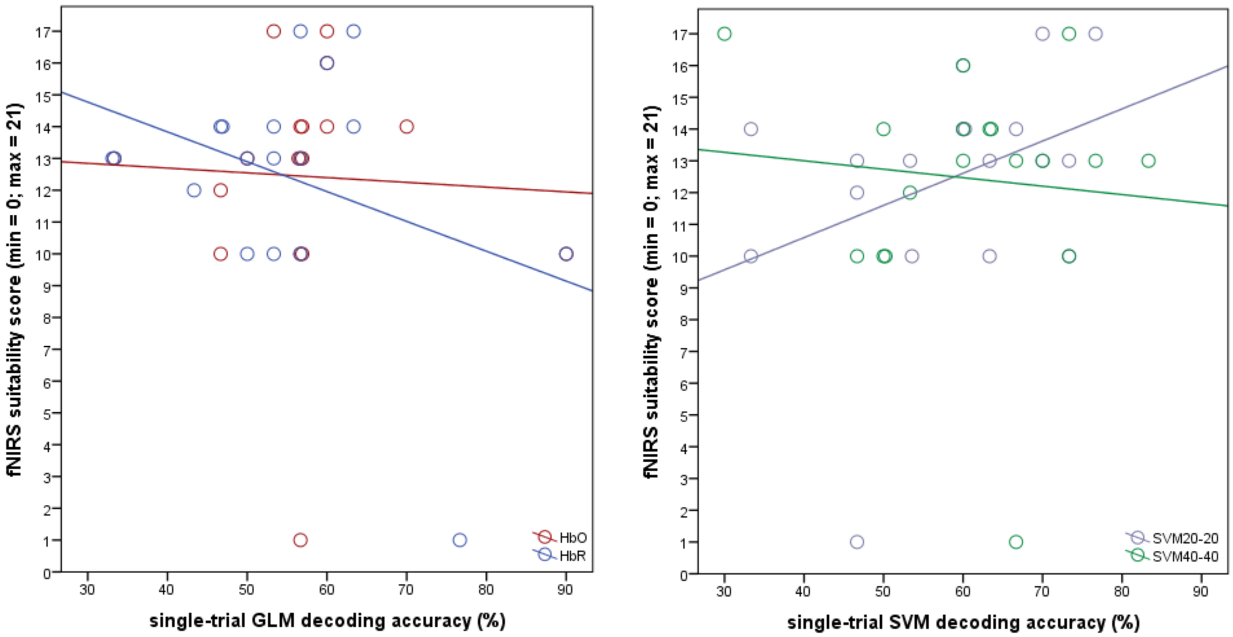

Figure S4: Scatterplots with single-trial decoding accuracy on the x-axis and fNIRS suitability score on the y-axis. Left panel: single-trials GLM decoding accuracy in function of fNIRS suitability score, grouped by data type (red circle = HbO; blue circle = HbR). The solid lines are linear regression lines per data type group. Right panel: single-trials SVM decoding accuracy in function of fNIRS suitability score, grouped by classifier training (grey circle = SVM20-20; green circle = SVM40-40).

The solid lines are linear regression lines per data type group (non-significant). Note that higher suitability scores on the y-axis indicate possibly less suitable participants for fNIRS measurements.
